# A cross sectional study to assess the sFlt-1:PlGF ratio in pregnant women with and without preeclampsia

**DOI:** 10.1186/s12884-019-2399-z

**Published:** 2019-07-25

**Authors:** Vivek Pant, Binod Kumar Yadav, Jyoti Sharma

**Affiliations:** 1 Department of Clinical Biochemistry, Institute of Medicine, Tribhuvan University Teaching Hospital, Kathmandu, Nepal; 2 Department of Gynecology and Obstetrics, Institute of Medicine, Tribhuvan University Teaching Hospital, Kathmandu, Nepal

**Keywords:** Preeclampsia, Soluble fms-like tyrosine kinase − 1 (sFlt-1), Placental growth factor (PlGF), Nepal

## Abstract

**Background:**

Preeclampsia is a multisystem disorder characterized by vascular endothelial malfunction occurring after 20 weeks of gestation. Placental soluble fms-like tyrosine kinase-1 (sFlt-1) is an antiangiogenic factor and placental growth factor (PlGF) is a potent angiogenic factor. The imbalance between these factors during placenta and fetal development has been shown to play a role in endothelial damage in preeclampsia.

Preeclampsia is the leading cause of maternal mortality in Nepal. This study was designed to compare the sFlt1:PLGF ratio in pregnant women with and without preeclampsia attending Tribhuvan University Teaching Hospital (TUTH).

**Method:**

An observational cross-sectional study was performed in the Gynecology and Obstetrics Department of TUTH involving forty-four subjects with preeclampsia and forty-four age- and gestational-week-matched normal pregnant subjects as controls. Blood pressure, urinary protein levels, serum sFlt-1 levels, serum PlGF levels and the sFlt-1:PlGF ratio was compared in both the cases and control. The concentrations of sFlt-1 and PlGF were measured with commercially available ELISA kits. SPSS ver. 20.0 was used to analyze the data.

**Results:**

There was no significant difference in age or gestational age in either study group. The ratio of the sFlt-1 and PlGF concentrations was significantly higher in women with preeclampsia (31.6 ± 9.6) than in the controls (3.2 ± 1.3). Likewise, diastolic blood pressure was significantly associated (*p*-value 0.000), whereas the severity of proteinuria was not associated (p-value 0.773) with the sFlt-1:PlGF ratio in women with preeclampsia. The significantly higher ratio (35.51 ± 8.1 versus 25.4 ± 8.7) was found in women with preeclampsia who developed complications than the group of women with preeclampsia who did not develop complication.

**Conclusion:**

The sFlt-1:PlGF ratio is significantly higher in Nepalese women with preeclampsia than in normal controls and this finding can be applied for further planned clinical trials.

## Background

Preeclampsia is a multisystem disorder characterized by the new onset of hypertension (above 140/ 90 mmHg) and proteinuria (0.3 g in a 24 h urine sample) occurring after 20 weeks of gestation [1]. Preeclampsia is associated with significant morbidity and mortality in both the mother and fetus. The maternal complications that can occur during pregnancy are oliguric renal failure, oligohydramnios, placental disruption, eclampsia, HELLP (Hemolysis, elevated liver enzyme and low platelet syndrome) and preterm labour [1]. Similarly, postpartum hemorrhage and puerperal sepsis can occur as complications after labour [1, 2]. Complications in the fetus and neonate are related to severity and duration of preeclampsia and include prematurity, fetal growth restriction, intrauterine fetal death and asphyxia [3]. Maternal symptoms of preeclampsia subside after delivery.

The underlying pathophysiology of preeclampsia involves endothelial dysfunction and vasospasm originating primarily in the placenta [4]. The abnormal development of blood vessels in placenta results in its under perfusion. This relative hypoxic condition in placenta causes release of antiangiogenic factors into the maternal blood circulation which leads to the alteration of maternal systemic endothelial function and causes hypertension [5].

Placental growth factor (PlGF), a glycoprotein that regulates blood vessel development is a member of vascular endothelial growth factor (VEGF) family [6]. VEGF interacts with their tyrosine kinase receptors which are of three types [7]. PlGF propagates signals through VEGF Receptor-1 (VEGF-R1) or Flt-1(fms like tyrosine kinase-1) but cannot bind VEGF Receptor − 2 (VEGF-R2) [6]. VEGF itself propagates signals through VEGF Receptor-2 (VEGF-R2) though it can bind to other receptors [6, 8]. PlGF enhances the activity of VEGF by competitively binding to VEGF-R1 and thus increasing VEGF interaction with VEGF R2, which is a potent stimulus for angiogenesis [6, 8].

The soluble variant of VEGF receptor-1 is also known as soluble fms like tyrosine kinase-1(sFlt-1), is an alternatively spliced variant of VEGF R1 which is induced by placental ischemia [9]. sFlt-1 inhibits angiogenesis by reducing free circulating levels of VEGF and PlGF [8]. This reduction of these pro angiogenic factors by sFlt-1 is brought by binding them in the circulation and preventing their interaction with receptors [8, 10]. VEGF receptor family shares the common feature of having extracellular domain consisting of seven immunoglobulins like domain and contains transmembrane and intracellular signaling domain [11]. As sFlt-1 is the spliced variant, it lacks the transmembrane and intracellular signaling domains [12]. This leads to free secretion of this anti angiogenic factor into the maternal circulation thus inhibiting the beneficial effects of proangiogenic factors on maternal endothelium [13].

The major share of maternal and perinatal morbidity and mortality in developing nation like Nepal can be attributed to preeclampsia/Eclampsia [14]. In clinical practice, the routine screening for preeclampsia is done by measuring blood pressure and quantification of protein in the urine. There is currently no standard practice for laboratory testing in early pregnancy that can predict the occurrence of preeclampsia. Rather, various preventive options are available for the high risk group such as administration of aspirin or calcium supplementation [15]. There is a need of sensitive laboratory test for screening preeclampsia because relying only on blood pressure measurement and proteinuria detection can be misleading. The main objective of the present study is to compare the serum sFlt-1:PlGF ratio in pregnant Nepalese women with and without preeclampsia; this ratio is also currently being tested in other parts of the world.

## Methods

An observational cross-sectional study was performed for a period of one year from August 2016 to August 2017. Singleton pregnant women ≥20 years of age with weeks of gestation ranging from 20 until delivery presenting with features of preeclampsia and the absence of exclusion criteria were recruited for this study by purposive sampling. The exclusion criteria were advanced maternal age (> 35 years, who might have essential hypertension), multiple fetuses, a history of chronic hypertension, a fetus with a congenital malformation incompatible with life as evidenced by ultrasonography (USG), the presence of disease causing proteinuria other than preeclampsia and placental insufficiency. Cases of preeclampsia in which the symptoms had started before 34 weeks of gestation were grouped as cases of early-onset preeclampsia. Similarly, cases of preeclampsia in which the symptoms had started after 34 weeks were grouped as cases of late-onset preeclampsia. Cases of preeclampsia were divided into mild and severe cases as defined by the American College of Obstetricians and Gynecologists 2012–2013 [1]. Forty-four age- and period-of-gestation (POG)-matched normal pregnant women served as controls; these were women attending regular antenatal visits in the Gynecology and Obstetrics OPD. Sample size was calculated by obtaining the prevalence of preeclampsia in the Gynecology and Obstetrics Department of our institute. Written consent was obtained from each participant.

The patients’ medical records were reviewed for the findings of USG, urinary protein level and blood pressure at the time of presentation. Five milliliters of venous blood was collected and centrifuged within 30 min; the serum was stored at − 40 °C until the analysis of serum sFlt-1 and PlGF. The sFlt-1 and PlGF levels of all samples were determined in a single run according to the manufacturer’s instructions for the research solid phase enzyme-linked immunosorbent assay (ELISA) kits purchased from Reddot Biotech Inc., Canada. The intra-assay coefficients of variation were 8.1% for serum sFlt-1 and 6.4% for PlGF.

Data were analyzed using SPSS for Windows version 20.0. All values are expressed as the mean +/− standard deviation. Data were tested for normality of distribution, and data that were not normally distributed were analyzed with nonparametric tests. Intergroup comparisons of continuous data were performed with Student’s t test or the Mann-Whitney U-test where appropriate. The serum sFlt-1:PIGF ratio is not increased in patients with preeclampsia was the hypothesis tested. Ethics approval was obtained from the Institutional Review Board (IRB) of the Institute of Medicine [Reference number – 263(6–11-E)^2^ 073/074].

## Results

The clinical details of the participants are shown in Table 1. Eighteen (40%) women with preeclampsia had early onset (< 34 weeks), and twenty-six (60%) women with preeclampsia had late onset (> 34 weeks). Twenty-three (52.27%) cases of preeclampsia were mild, and twenty-one (47.72%) cases were severe.

**Table 1:** Clinical details of participants.

Clinical details of participants	Preeclampsia (*N* = 44)	Normal pregnancy (*N* = 44)
Age (Mean ± SD) years	27.3 ± 3.1	26.28 ± 2.5
Period of gestation (Mean ± SD) weeks	35.1 ± 3.0	34.9 ± 3.2
Parity	21 (47.72%) multiparous	15 (34.09%) multiparous
	23 (52.27%) primiparous	29 (65.90%) primiparous

**Fig. 1:**
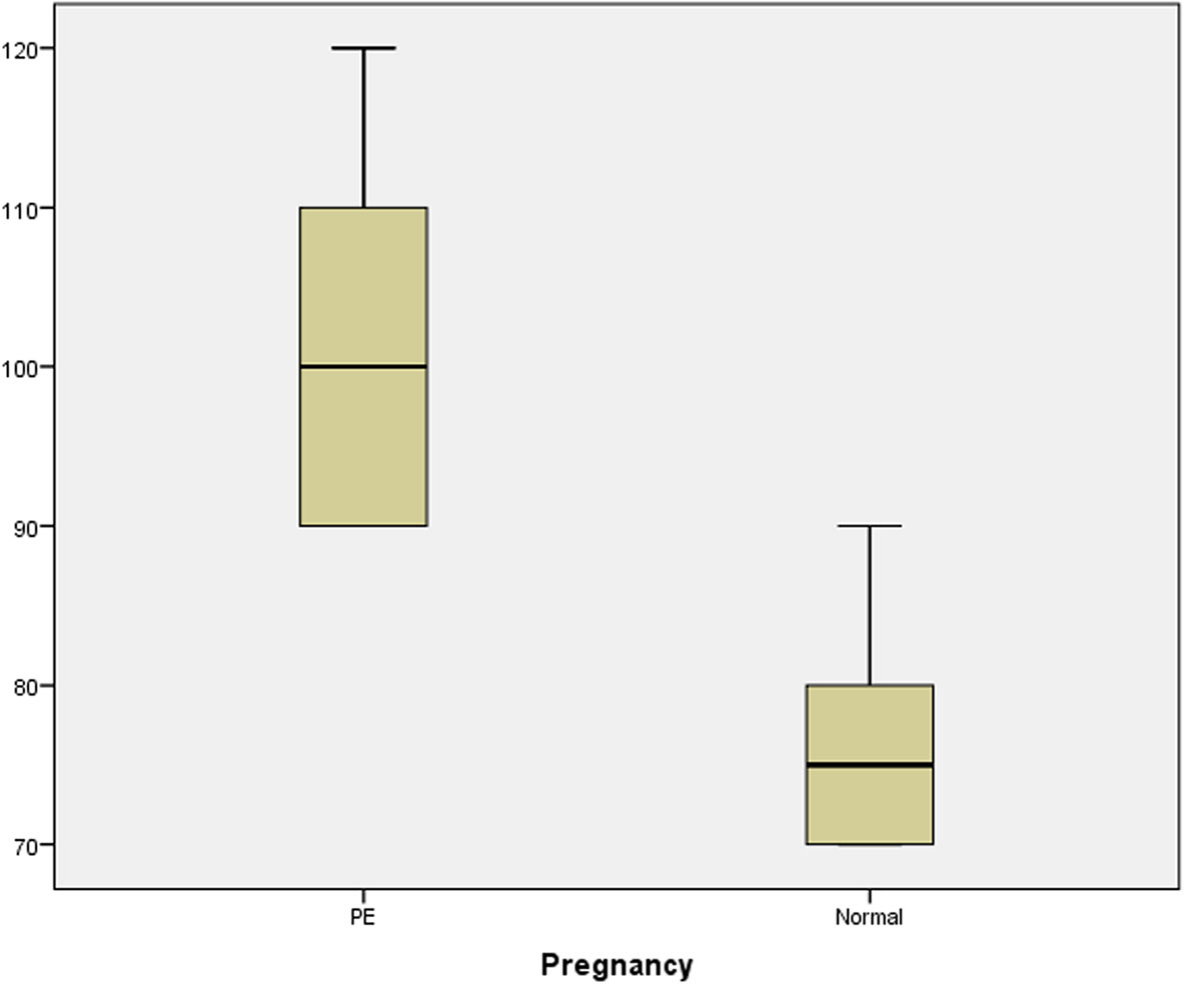
Box plot showing mean value of diastolic blood pressure in case and control

**Fig. 2:**
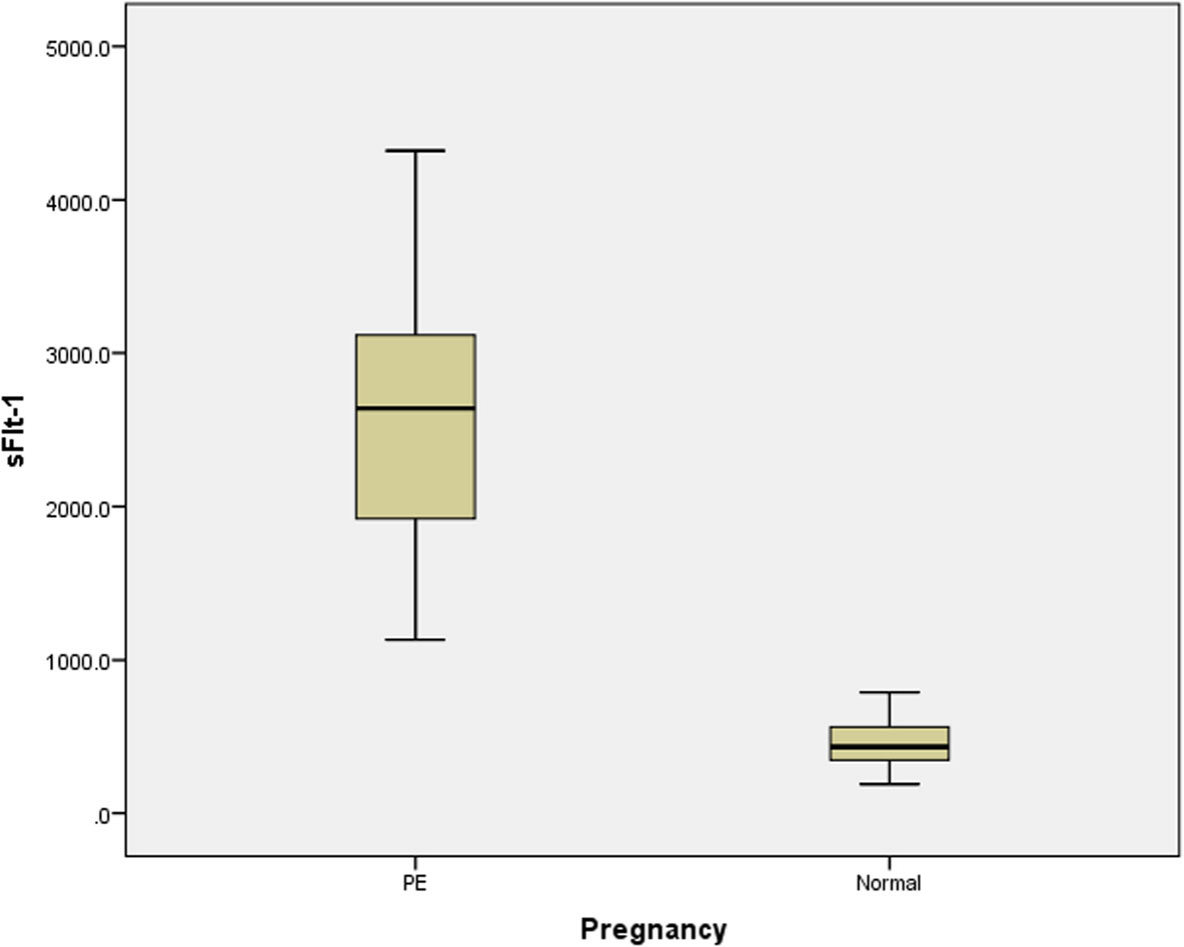
Box plot showing mean value of sFlt-1 in case and control

**Fig. 3:**
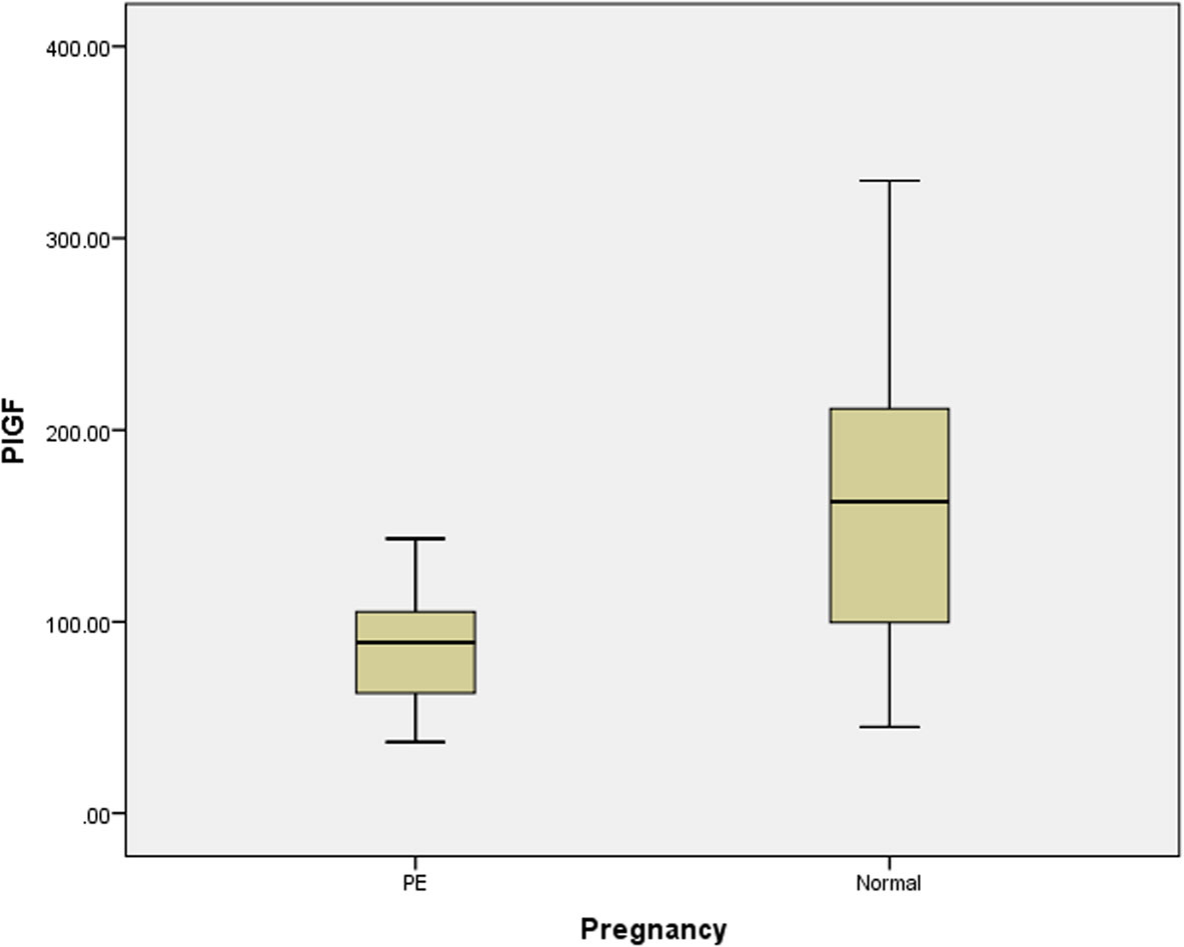
Box plot showing mean value of PlGF in case and control

**Fig. 4:**
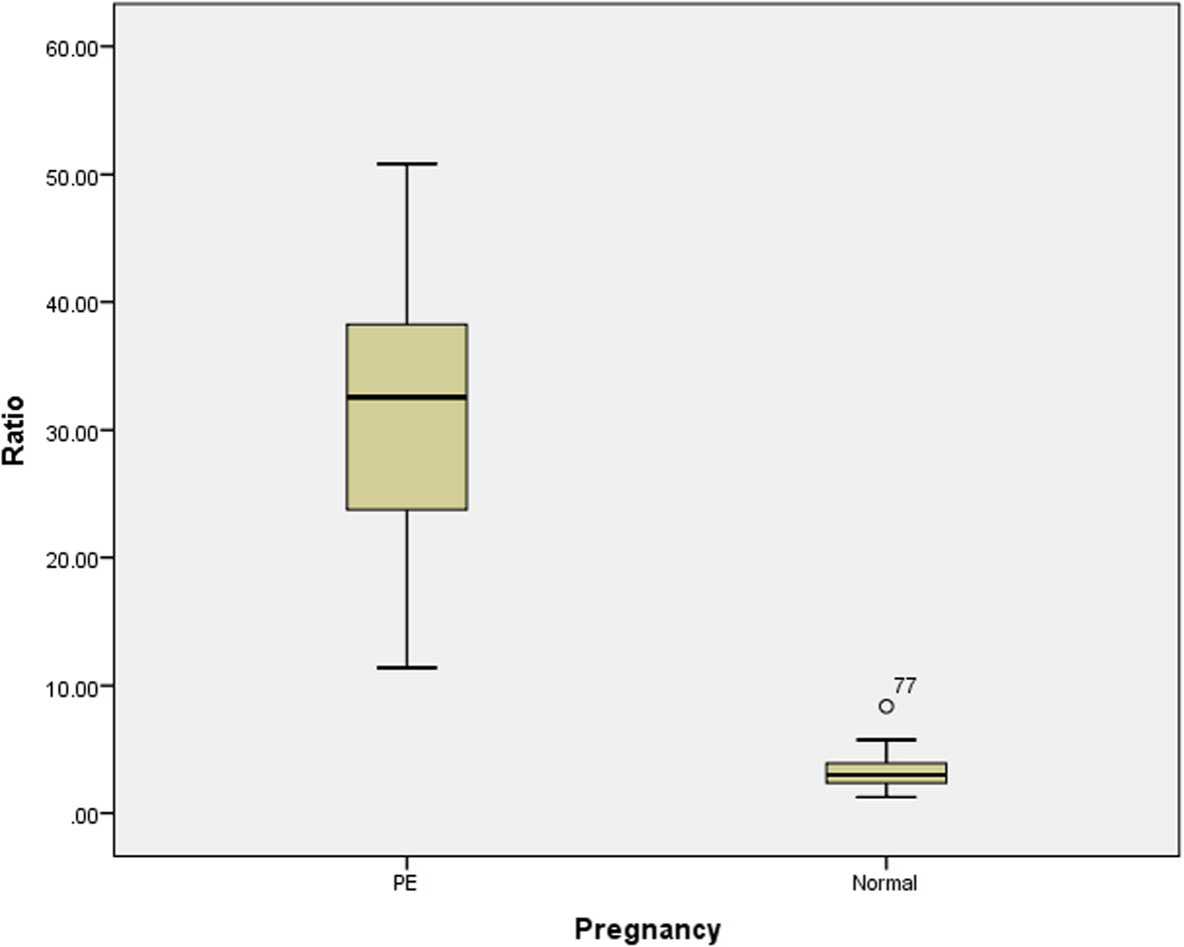
Box plot showing mean ratio of sFlt-1: PlGF in case and control

There was a significant difference in the mean values of DBP, sFlt-1, and PlGF and the sFlt-1:PlGF ratio between the cases and controls, as shown in Table 2 and Figs. 1, 2, 3, 4. Comparisons of the levels of sFlt-1 and PlGF and their ratio in mild vs. severe cases and early onset vs. late onset preeclampsia are shown in Table 3.

**Table 2:** Comparison of means of age, POG, sFlt-1, PlGF and sFlt-1:PlGF ratio in case and control

Parameters	Preeclampsia Mean ± SD	Normal Mean ± SD	*P*-value
Age	27.3 ± 3.1	26.3 ± 2.5	0.393
POG	35.3 ± 3.0	34.9 ± 3.2	0.875
sFlt-1	2575.50 ± 775.03	453.75 ± 156.24	< 0.001*
PlGF	86.31 ± 26.9	155.8 ± 63.89	< 0.001*
Ratio	31.6 ± 9.6	3.2 ± 1.3	< 0.001*
DBP	101.1 ± 9.4	76.3 ± 5.6	< 0.001*

*Abbreviation*: POG- period of gestation, DBP- Diastolic blood pressure, sFlt-1 – soluble Fms like tyrosine kinase-1, PlGF- Placental growth factor

* significant at < 0.001

**Table 3:** Comparison of sFlt-1, PlGF and ratio in mild/severe and early/late onset PE

Preeclampsia	sFlt-1 (mean)	PlGF (mean)	Ratio (mean)
Severe PE	2885.16	92.56	31.51
Mild PE	2292.76	80.60	31.72
P-value	0.010*	0.143	0.945
Early onset	3017.11	95.24	32.79
Late onset	2269.76	80.12	30.81
P-value	0.001*	0.050*	0.945

* significant at 0.05

The numbers of patients with trace, 1+, 2+, 3+, and 4+ proteinuria were seven (7.9%), fourteen (15.7%), ten (11.2%), ten (11.2%) and three (3.4%), respectively. There was no significant association found (*P* value 0.773) between the degree of proteinuria and the ratio of sFlt-1:PlGF in patients with preeclampsia.

**Fig. 5:**
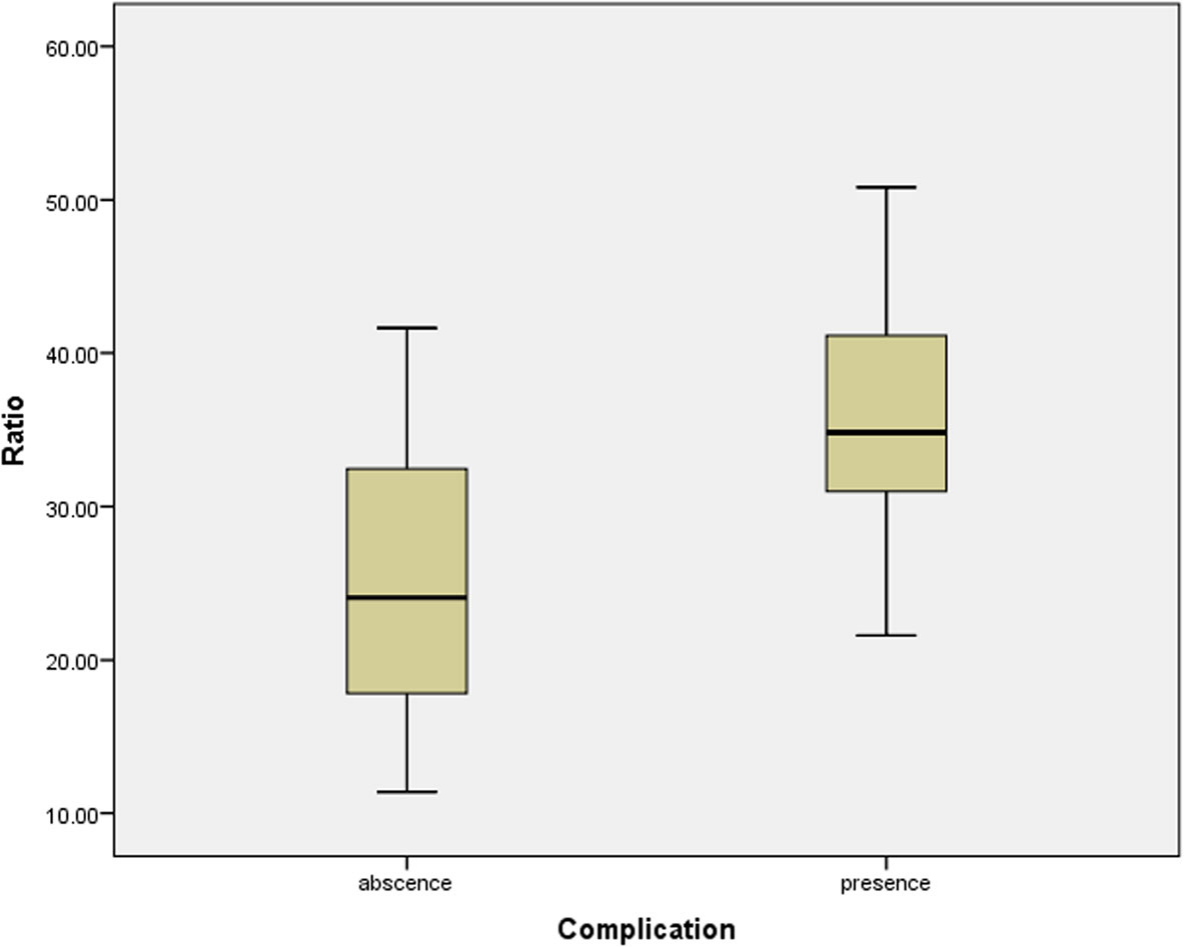
Box plot showing mean ratio of sFlt-1: PlGF in preeclampsia cases with absence or presence of complication

Patients with preeclampsia were divided into two groups based on the presence or absence of complications. When the mean sFlt-1:PlGF ratio was compared between these groups, a significantly higher ratio (35.51 ± 8.1 versus 25.4 ± 8.7) was found in the group with complications. (Fig. 5) The types of complications and their frequencies in patients with preeclampsia are shown in Table 4.

**Table 4:** Types of complication and its frequency in cases of preeclampsia

Complication	Frequency	Percentage
Abruptio Placentae	1	2.27%
Birth Asphyxia	1	2.27%
Persistent thrombocytopenia	1	2.27%
IUFD	1	2.27%
Elevated Liver enzymes	2	4.54%
Eclampsia	4	9.09%
IUGR	8	18.18%
Low birth weight	9	20.45%

## Discussion

The relative overproduction of sFlt-1 than VEGF by placental tissue in preeclampsia is responsible for various clinical manifestations seen in preeclampsia [16]. Studies have shown that this spliced variant decreases PlGF and contributes to endothelial dysfunction, hypertension, and proteinuria observed in women with preeclampsia [13, 17]. To the best of our knowledge, this is the first study in Nepal comparing the levels of sFlt-1 and PlGF in pregnant women with and without preeclampsia.

In our study, the serum PlGF level was decreased (86 ± 26.9 pg/ml versus 155 ± 63.89 pg/ml) and the serum sFlt-1 level was elevated (2575 ± 775.03 pg/ml versus 453 ± 156.24 pg/ml) in the pregnant women with preeclampsia than the pregnant women without preeclampsia. Similarly, the ratio of sFlt-1/PlGF in the pregnant women with preeclampsia was significantly higher than that in the control group. In a study by Levine et al., a higher mean sFlt-1 value of 4382 pg/ml was reported in women with preeclampsia compared to value of 1643 pg/ml in the control group [18]. In the two separate studies by Kim et al. [19] and Gurnadi JI et al. [20], elevated serum concentrations of sFlt-1 in women with preeclampsia was found.

In our study, the serum sFlt-1 concentration was significantly higher in women with severe preeclampsia than in those with mild preeclampsia (*p* < 0.001). It has been found that the elevation of the sFlt-1 concentration reduces the PlGF concentration in severe preeclampsia, and this occurs before the onset of clinical symptoms [21, 22]. Chaiworapongsa et al., reported that the increasing concentration of sFlt-1 correlates with severity of disease [23]. Similarly, the severity of preeclampsia is also found positively correlated with the increased urinary output of sFlt-1 at the time of clinical manifestation [24]. These findings suggest that periodic monitoring of sFlt-1 levels in cases of preeclampsia can be important in the early identification of the onset of severity of preeclampsia.

In the present study, the degree of proteinuria was not significantly associated with the ratio of sFlt-1:PlGF in women with preeclampsia (*p*-value 0.773). In this study, a significant number of women with preeclampsia were the hospitalized patients to whom antihypertensive drugs and intravenous fluids were already administered. This might have reduced the severity of proteinuria in these patients, which may explain the insignificant association of proteinuria with the ratio of sFlt-1:PlGF. Proteinuria was estimated by the dipstick method in our study, which is a more qualitative measure. The 24-h protein or urine/protein creatinine ratio is more reliable, but it could not be performed in our patients. Most patients in this study had already manifested preeclamptic symptoms. Proteinuria was present in all patients with preeclampsia, and the degree of proteinuria was higher in severe than mild cases of preeclampsia. This finding is similar to the findings of Barton et al., who reported that proteinuria generally increase as preeclampsia progressed but that increased urinary protein excretion may be a late finding [25]. In a recently published study by Dong et al.*,* the severity of preeclampsia was higher when proteinuria was above 0.3 g/L [26].

In our study, the diastolic blood pressure was significantly associated with the sFlt-1:PlGF ratio in the group with preeclampsia. This finding is consistent with those of previous studies. Reiss et al. found that both the systolic and diastolic blood pressure values were significantly higher in the first trimester for women who later developed preeclampsia than for those who did not [27]. Various theories have been put forward regarding the etiology of hypertension in preeclampsia. It has been suggested that the circulating sFlt-1 concentration in preeclamptic patients antagonizes physiologic dilatation of blood vessels, thus contributing to hypertension [13]. The current medical practice for diagnosing preeclampsia, which is also a simple and inexpensive method of screening in low-resource settings such as ours, is the measurement of blood pressure and proteinuria. While the most recent diagnostic markers, such as the sFlt-1:PlGF ratio, are currently used in other parts of the world, we are still lagging behind in the application of routine screening tests. The recent trends in Nepal showed an increased use of antenatal care; however, 17% of pregnant women receive no antenatal care (ANC), and among those who do, routine screening is often not carried out [28]. Thus, the findings of this study emphasize the importance of measuring blood pressure.

In the present study, the concentrations of sFlt-1 and PIGF were significant higher in patients with early-onset preeclampsia (< 34 weeks) than in those with late-onset PE (> 34 weeks). A similar finding was reported in a study by Chaiworapongsa et al., in which the sFlt-1 concentrations were higher in women with early-onset (< 34 weeks) preeclampsia [23]. Wikström et al. reported that women with early-onset PE had 43 times higher median plasma sFlt-1 levels and 21 times lower median plasma PIGF levels than controls (*p* < 0.001) [29]. It has been found that late onset preeclampsia is more prevalent than early onset preeclampsia [30]. The lower concentrations of sFlt-1 and PlGF in late-onset preeclampsia are likely due to its non-placental origin because it occurs due to pre-existing maternal endothelial dysfunction [31].

This is the first study in the Nepalese population reporting increased sFlt1:PlGF ratio in women with preeclampsia than in normal controls. Despite being the leading cause of high maternal mortality rate in Nepal, the clinical study in preeclampsia is limited. Various prospective clinical studies could be done in pregnant women in Nepal based on findings of our study. For example, Ziesler et al. in their study suggested that the sFlt-1:PlGF ratio may be useful as a negative predictive test in clinically suspected cases [32]. Our finding is important for future clinical studies in suspected cases of preeclampsia. It is also worth correlating these markers with complications of preeclampsia in mother and fetus.

The limitation of our study is that the samples were taken from confirmed cases of preeclampsia at one point of time rather than the prospective study in the suspected cases of preeclampsia. The next limitation was the use of the dipstick method for determination of proteinuria and the use of research ELISA kits, which are less sensitive.

## Conclusion

In this study, the sFlt-1 level was increased and the PlGF level was decreased in Nepalese women with preeclampsia compared to normal pregnant women. Similarly, the ratio of sFlt-1:PlGF was higher in women with preeclampsia than those without preeclampsia. The results of our study will be helpful for further planned trials in clinically suspected cases of preeclampsia in Nepal.

## Data Availability

The data that support the findings of this study are available from the Institutional Research Board of the Institute of Medicine, but restrictions apply to the availability of these data, which were used under license for the current study and so are not publicly available. Data are, however, available from the authors upon reasonable request and with permission of the Institutional Research Board of the Institute of Medicine.

## Abbreviations

ACOG: American College of Obstetricians and Gynecologists
ANC: Antenatal Care
DBP: Diastolic Blood pressure
E: Eclampsia
ELISA: Enzyme Linked Immunosorbent Assay
HELLP: Hemolysis Elevated Liver enzyme and low platelet
IOM: Institute of Medicine
IRB: Institutional research board
OPD: Out patient department
PE: Preeclampsia
PlGF: Placental Growth Factor
POG: Period of Gestation
sFlt-1: Soluble fms like tyrosine kinase-1
SPSS: Statistical package for social science
TUTH: Tribhuvan university teaching hospital
USG: Ultrasonography
